# Supplementary material for: Inflammatory factors and risk of lung adenocarcinoma: a Mendelian randomization study mediated by blood metabolites
Source: Front Endocrinol (Lausanne). 2024 Aug 27;15:1446863. doi: 10.3389/fendo.2024.1446863 (PMC11384989; doi:10.3389/fendo.2024.1446863)
Supplement: Supplementary file 1 [file DataSheet1.zip › supplementary files/S2/GCST90200992/sensitivity-analysis.pdf]

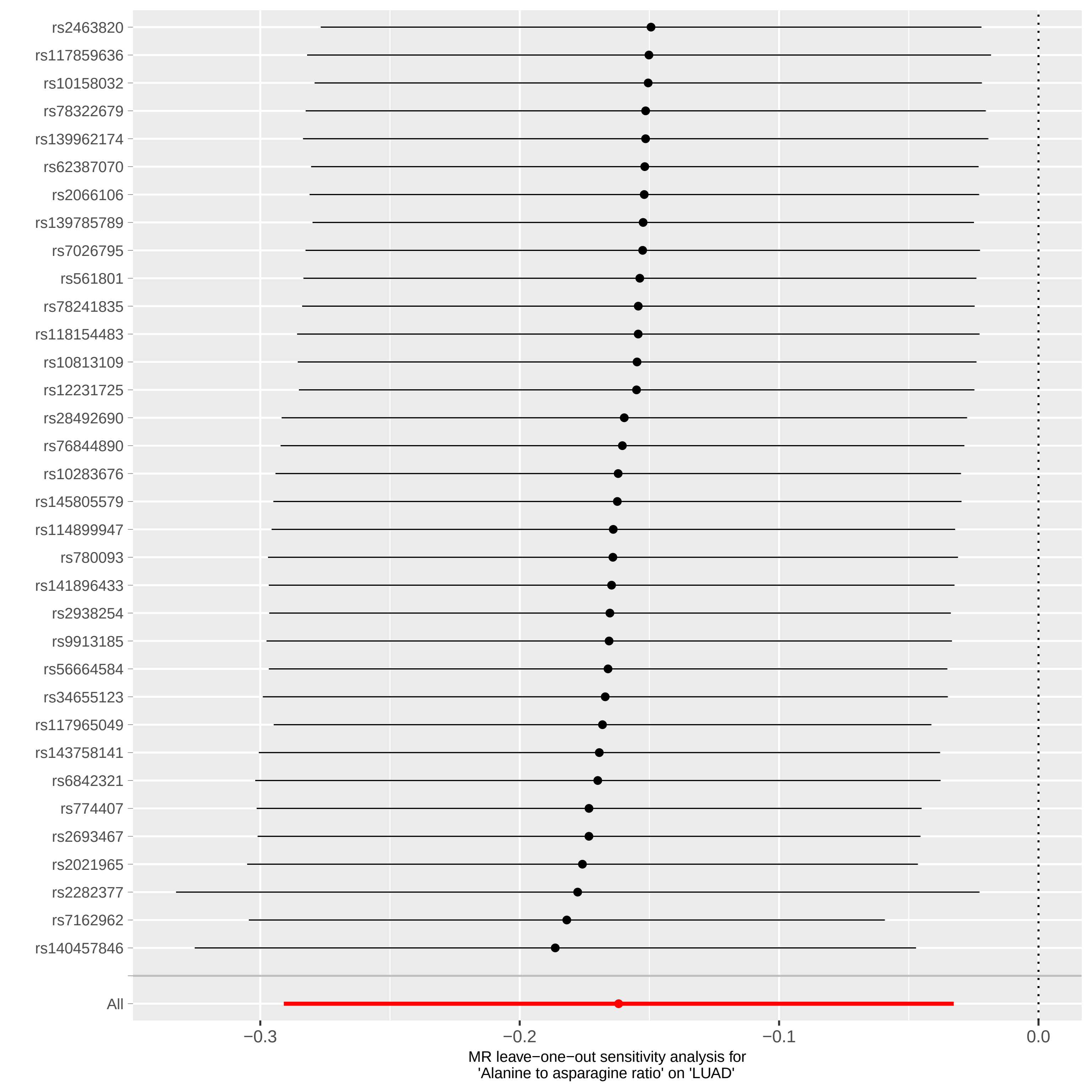
rs2463820
rs117859636
rs10158032
rs78322679
rs139962174
rs62387070
rs2066106
rs139785789
rs7026795
rs561801
rs78241835
rs118154483
rs10813109
rs12231725
rs28492690
rs76844890
rs10283676
rs145805579
rs114899947
rs780093
rs141896433
rs2938254
rs9913185
rs56664584
rs34655123
rs117965049
rs143758141
rs6842321
rs774407
rs2693467
rs2021965
rs2282377
rs7162962
rs140457846
All
−0.3
−0.2
−0.1
0.0
MR leave−one−out sensitivity analysis for
'Alanine to asparagine ratio' on 'LUAD'
